# Comments on Post-Publication Discussion of “Evaluation of a Gene Expression Profiling Assay in Primary Cutaneous Melanoma”

**DOI:** 10.1245/s10434-022-11651-9

**Published:** 2022-04-05

**Authors:** Robert W. Cook, Matthew S. Goldberg

**Affiliations:** 1 Castle Biosciences, Inc., Friendswood, TX USA; 2 Icahn School of Medicine at Mount Sinai, New York, NY USA

To the Editors

We thank the *Annals of Surgical Oncology* (*ASO*) editors for posing substantive questions in “Post-Publication Discussion: Invitation for Reply” and the article authors of “Post-Publication Discussion: In Reply” for attempting to address those queries.^1,2^ However, the responses address only a small subset of the articulated concerns and highlight the importance of understanding appropriate methodological and statistical practices that should be implemented when evaluating prognostic testing. We initiated the post-publication discussion by submitting a request for erratum and, potentially, retraction of “Evaluation of a Gene Expression Profiling Assay in Primary Cutaneous Melanoma”.^3^ Our request prompted a review of the original, published manuscript that subsequently identified methodological concerns that editors deemed necessary to publish in *ASO*.^1^ Specifically, author responses included methodological changes to remove recognized biases and included revisions to the study that substantially reduced the cohort size (15.5% reduction; *n* = 56) and total recurrences (24.5% reduction; *n* = 12).^2^ Importantly, these changes altered the observed statistical findings from multivariate analysis without a concomitant adjustment to author conclusions. Considering these points, we find the authors’ responses to editorial questions to be incomplete and incompatible with the original published manuscript. In the context of the authors’ confirmation of methodological omissions, we request further scrutiny of the original published manuscript, with publication of additional supporting data or an erratum, at a minimum.

We are concerned that the cohort changes described in the post-publication response (i.e., removing tested patients from the analysis) exacerbate two of the original article limitations: the “somewhat limited” overall statistical power and the “limited follow-up time”.^3^ Our assessment is that the authors’ approach to the inclusion/exclusion of cases, with limited events and limited follow-up time, resulted in an underpowered multivariate analysis to appropriately detect significance (or insignificance) of the tested prognostic variables. Statistical assumptions that form the basis of power calculations were omitted from both the original article and the authors’ reply. The authors’ awareness of the statistical considerations is further complicated by the fact that they are unable to provide (and, in fact, “do not have ready access to”^2^) the number of cases and events included in univariate and multivariate analyses; this information is easily accessible from statistical programs used to conduct multivariate regression analyses.

We do appreciate the transparency demonstrated by the authors in updated Tables 1 and 2 from their reply, but we question the omission of similar tables for the updated multivariate analysis that excluded all retrospectively tested patients. Further, although the cohort described in the original article had a median follow-up period of 15.3 months, we assume that the median follow-up period for the revised cohort would be substantially shorter, given the exclusion of the cases retroactively ordered between 2013 and 2015. Readers are not provided with that information in the reply and gain no additional clarity about the original article limitations specified by the study authors.

Another nonmethodological concern with the original article was the lack of alignment with the central dogma for currently considered pathologic factors for melanoma [e.g., sentinel lymph node biopsy (SLNB) status, Breslow thickness (BT)]. Specifically, in the original article discussion, the authors characterized their findings as supportive of AJCC staging despite author admission that the insignificance of SLNB as a prognostic marker in the original multivariate analyses constituted an “unexpected finding”.^3^ More alarming is the finding for recurrence-free survival (RFS) that SLNB gains significance while BT loses significance as a prognostic factor when retroactively ordered cases are removed. We sincerely doubt that the *ASO* editors, *ASO* readership, and the article authors themselves question the prognostic significance of SLNB or BT.^4^ The discordant results suggest a flaw in the methods implemented by the authors and conflict with AJCC staging.^5^ SLNB and BT are significant pathologic factors that provide independent prognostic information, and the changing status of these variables in different iterations of data analysis calls into question the integrity of the statistical methodology and undermines all conclusions drawn. In this context, consideration should be given to the authors’ conclusions about 31-gene expression profile (GEP) testing, which contradict all retrospective and prospective validation studies to date.^6–8^

If, as we strongly suspect, this study reports an underpowered multivariate analysis, it is impossible to draw any conclusions about factors not found to be significant in a model as the lack of statistical power may obscure relationships known to be significant. The authors have made interpretive leaps about the relative value of 31-GEP testing not supported by their study design and analysis plan, while simultaneously omitting interpretive critiques that could be similarly applied to the reported nonsignificance of various AJCC pathologic factors (SLNB in original manuscript and BT in the author responses). Those who accept the findings of nonsignificance of GEP in this poorly defined cohort must also wrestle with the simultaneous conclusion that SLNB or BT are nonsignificant risk stratification factors in these patients.
